# Supplementary material for: Scalable Generation of Mesenchymal Stem Cells and Adipocytes from Human Pluripotent Stem Cells
Source: Cells. 2020 Mar 13;9(3):710. doi: 10.3390/cells9030710 (PMC7140720; doi:10.3390/cells9030710)
Supplement: Supplementary file 1 [file cells-09-00710-s001.zip › cells-703329-supplementary.pdf]

Supplementary Figures

Figure S1

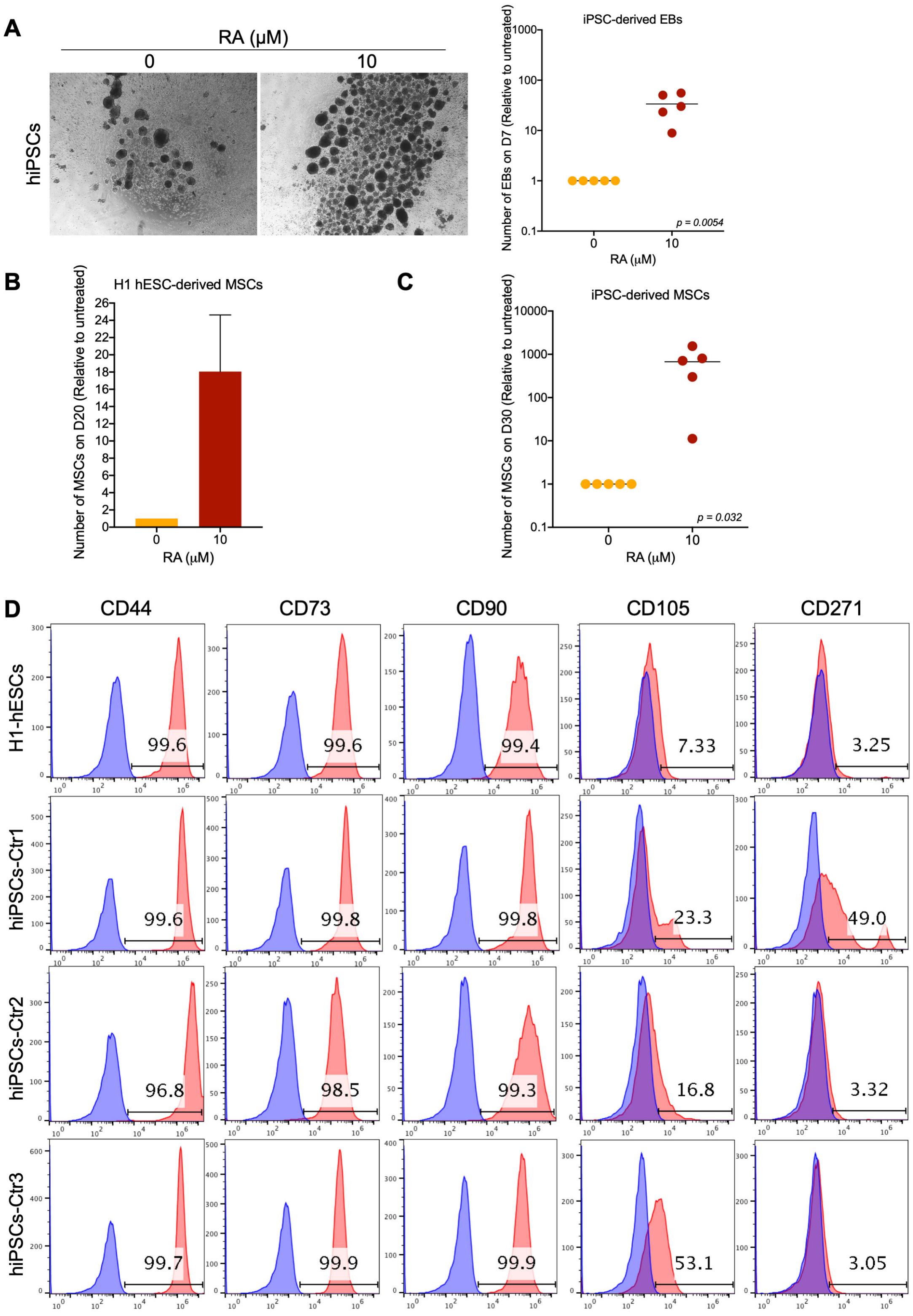

Supplementary Figure 1. Effect of short-term treatment with high RA concentration on the efficiency of differentiation of human iPSCs into EBs and MSCs. Human iPSCs (clones generated from three different healthy donors) were differentiated into MSCs as described in Fig 1A of this manuscript without or with treatment with 10 µM RA. A. On day 7 of differentiation, EBs were photographed and counted. Images (10x magnification) for representative hiPSC-derived EBs are shown. The scatter plot presents the individual distributions (dots) and the means (bars) of the fold-increases in EB number (relative to RA untreated condition) on D7 of differentiation of four iPSC clones generated from the PBMCs of three different healthy donors (one to two different clones per donor). B. Scatter plot presenting the fold-increase (relative to RA untreated condition) in the number of MSCs obtained between D25 and D30 of differentiation of three iPSC clones derived from the PBMCs of three healthy donors. Two different representative differentiations were conducted for each clone and presented in the scatter plot. C. Effect of EB treatment with 10 µM RA on the number of MSCs generated from H1-hESCs.The bar graph presents the means ± SD for two independent experiments. D. Flow cytometry analysis of the expression of the MSC markers CD44, CD73, CD90, CD105 and CD271 in the MSCs generated from H1-hESC- and different iPSC-derived EBs treated with 10 µM RA. The histograms presented indicate the percentage of marker-positive cells and are representative for two independent experiments.

Figure S2

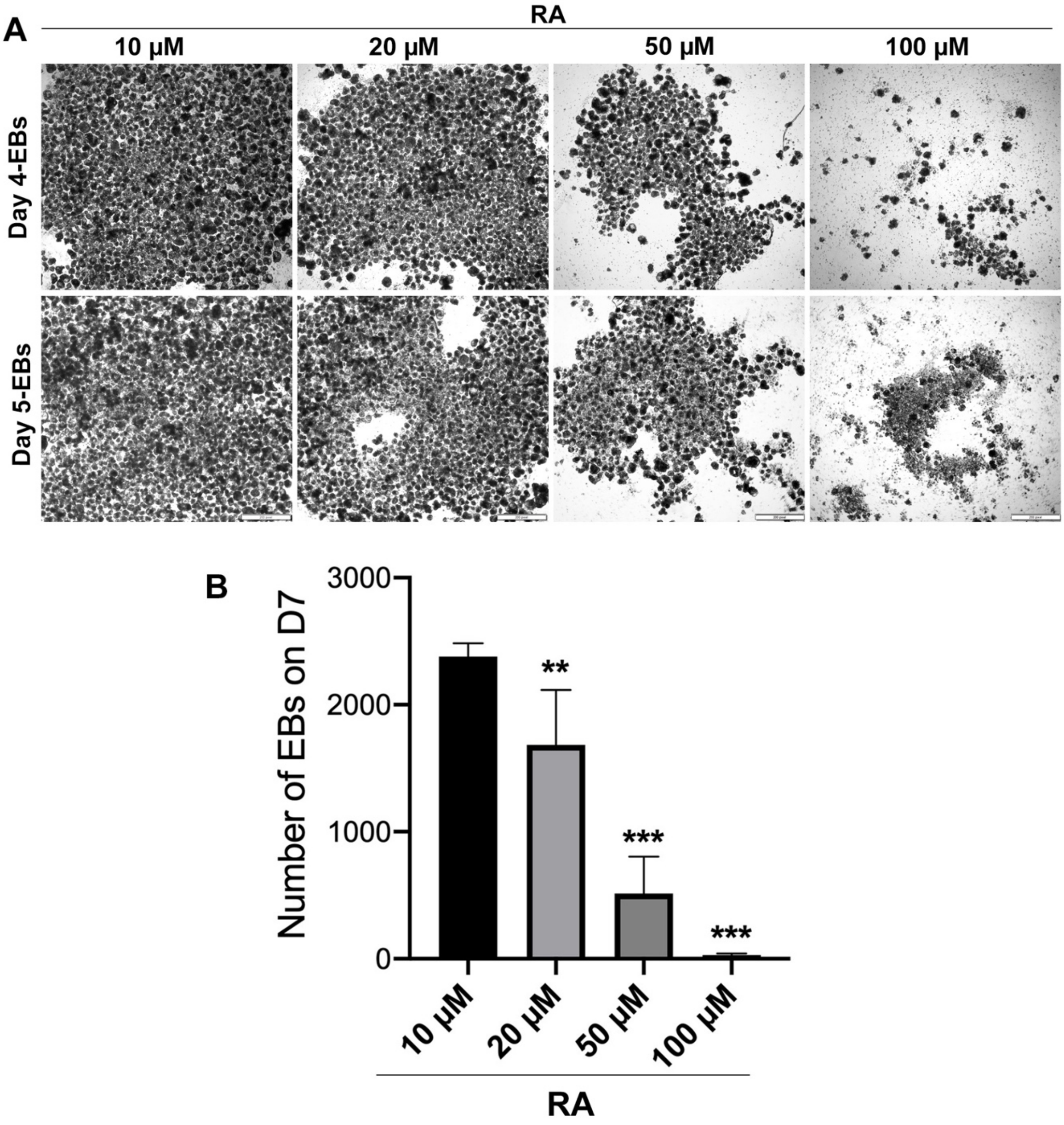

Supplementary Figure 2. The effect of high concentrations of RA on EB formation. (A) Representative images of H9 hESC-derived EBs showing the effect of high concentrations of RA (10, 20, 50, and 100 μM) on EB formation at D4 (day 4) and D5 (day 5) of differentiation. (B) The EBs were quantified at D7 of differentiation. The results presented in the bar graph are means ± SD for two independent experiments. *$p<0.05$ and **$p<0.01$ versus EBs treated with 10 μM.

Figure S3

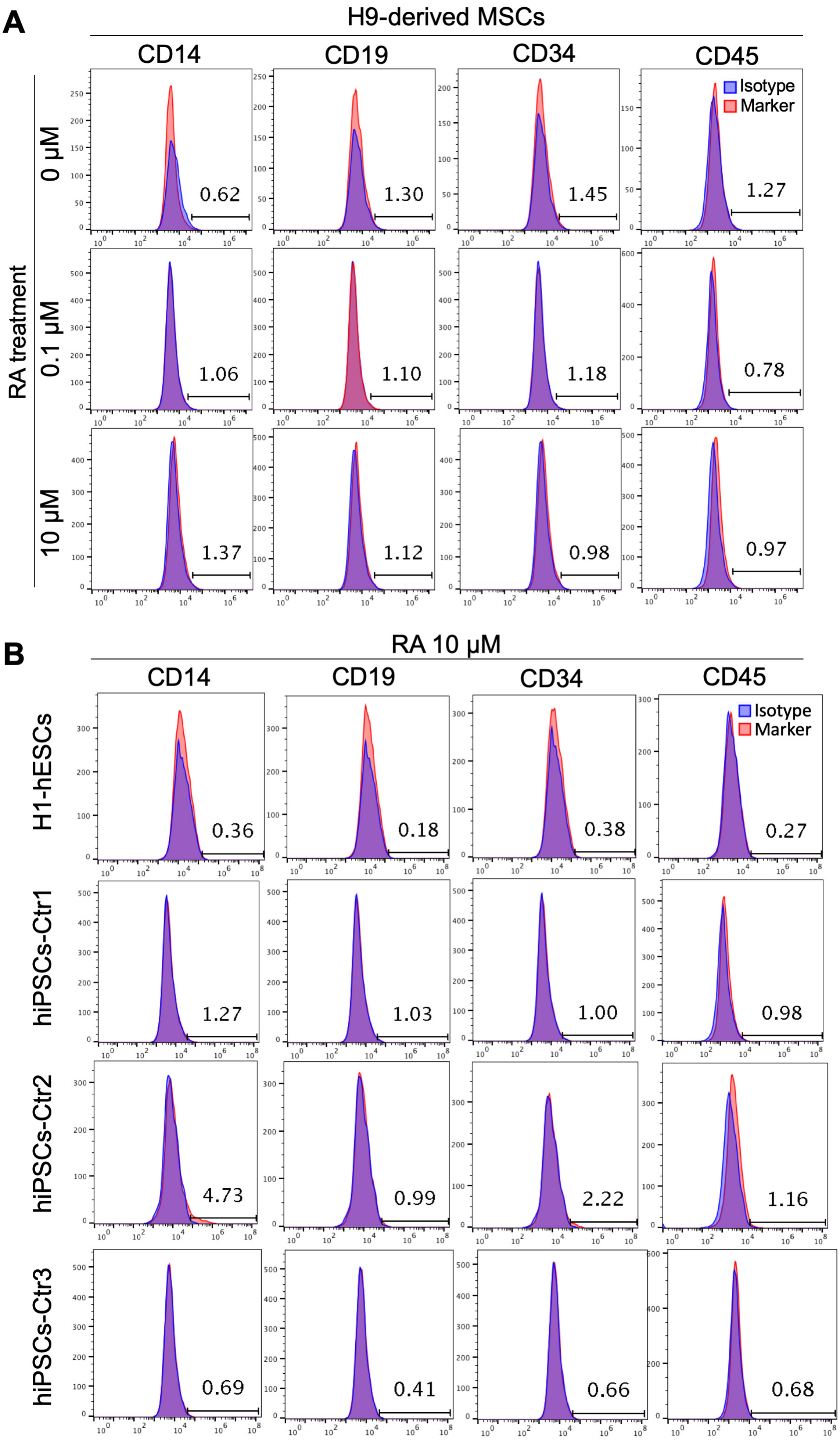
A
H9-derived MSCs
CD14
CD19
CD34
CD45
Isotype
Marker
RA treatment
0 μM
0.1 μM
10 μM
0.62
1.30
1.45
1.27
1.06
1.10
1.18
0.78
1.37
1.12
0.98
0.97
B
RA 10 μM
CD14
CD19
CD34
CD45
Isotype
Marker
H1-hESCs
hiPSCs-Ctr1
hiPSCs-Ctr2
hiPSCs-Ctr3
0.36
0.18
0.38
0.27
1.27
1.03
1.00
0.98
4.73
0.99
2.22
1.16
0.69
0.41
0.66
0.68

Supplementary Figure 3. Absence of hematopoietic markers' expression in the PSC-derived MSCs. (A) H9 hESC-derived EBs were treated or not with different RA concentrations and then differentiated into MSCs. The expression of the hematopoietic markers CD14, CD19, CD34 and CD45 was assessed on D20 of differentiation by flow cytometry. The histograms presented indicate the percentage of marker-positive cells and are representative from five-independent experiments. (B) H1 hESC-derived and healthy donor iPSC-derived EBs treated with 10 μM RA and differentiated into MSCs were assessed for the expression of the hematopoietic markers CD14, CD19, CD34 and CD45 on D20 of differentiation by flow cytometry. The histograms presented indicate the percentage of marker-positive cells and are representative from two-independent experiments.

Figure S4

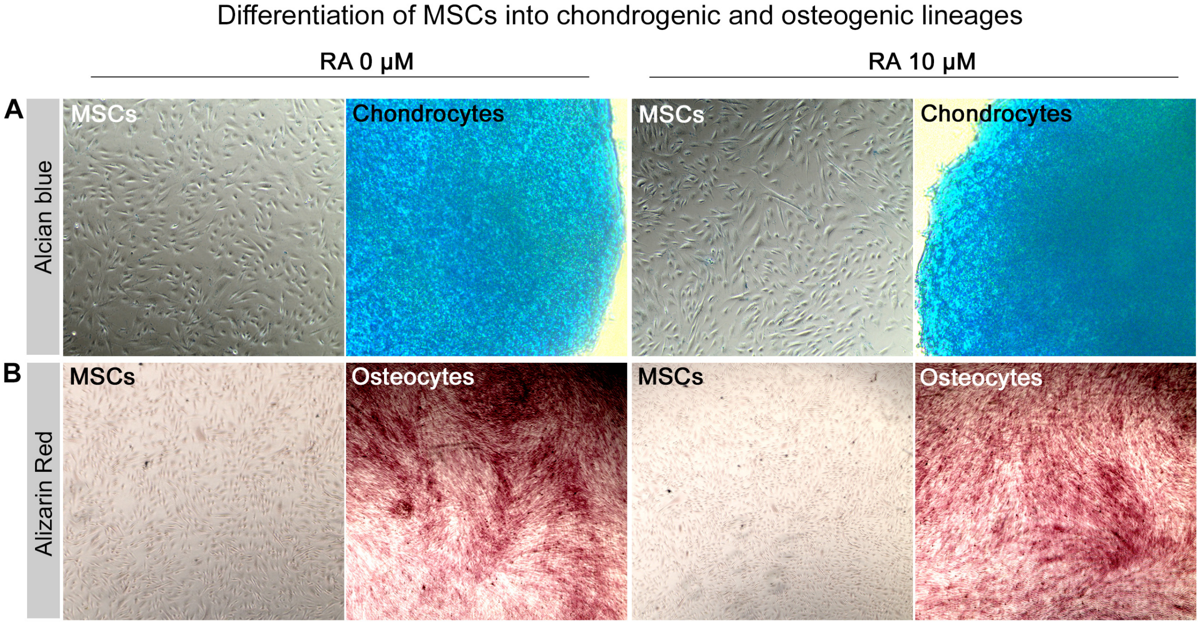

Supplementary Figure 4. Differentiation of hPSC-derived MSCs into chondrocytes and osteocytes. MSCs derived from EBs treated or not with 10 μM were differentiated into chondrocytes and osteocytes. Representative images show staining of differentiated cells with alcian blue (A) and alizaran red (B), indicating their successful differentiation into chondrocytes and osteocytes. Undifferentiated MSCs were used as negative control.

Figure S5

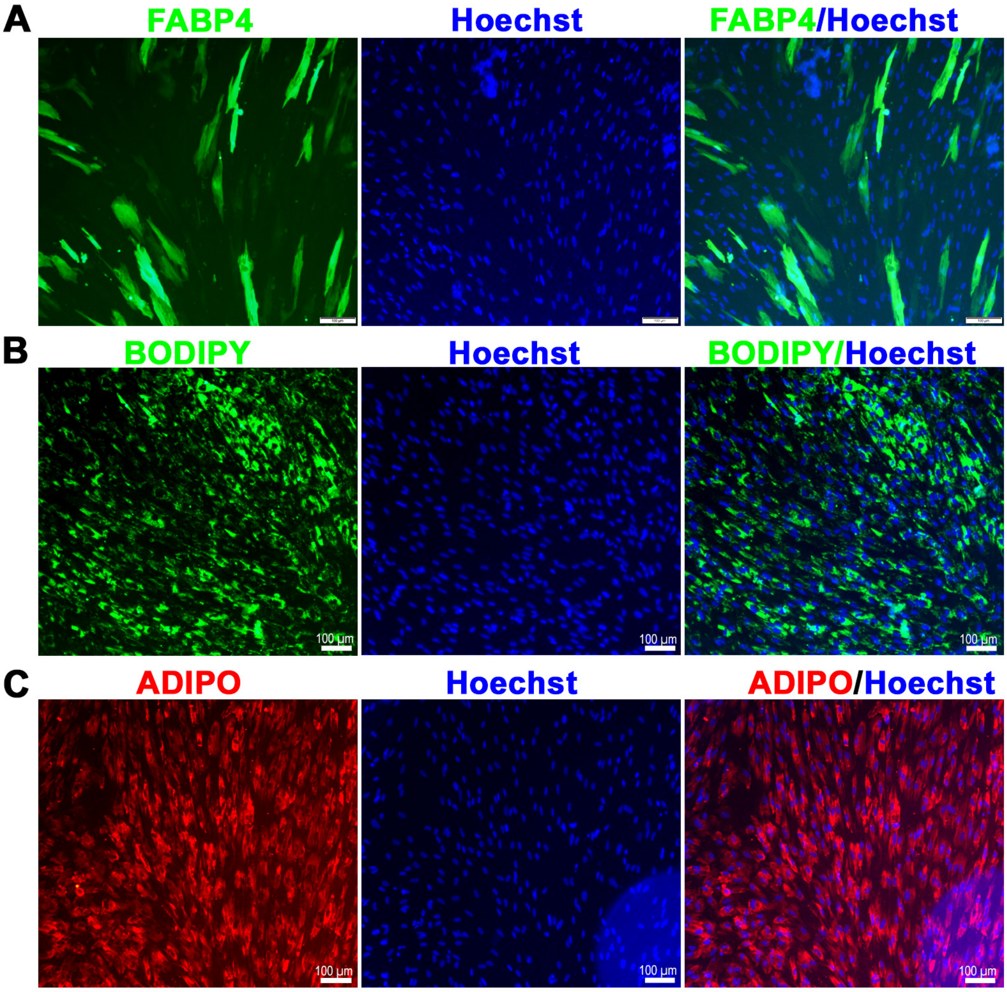

Supplementary Figure 5. hiPSC-derived MSCs display an enhanced differentiation potential into adipocytic lineage. MSCs obtained from hiPSC-derived EBs treated with 10 µM RA were differentiated into adipocytes using protocol 1. Representative immunofluorescence images showing the expression of FABP4 (A), BODIPY (B), and adiponectin (ADIPO) in the adipocytes derived from the MSCs obtained from hiPSC-Ctr1-derived EBs treated with 10 µM RA.

Figure S6

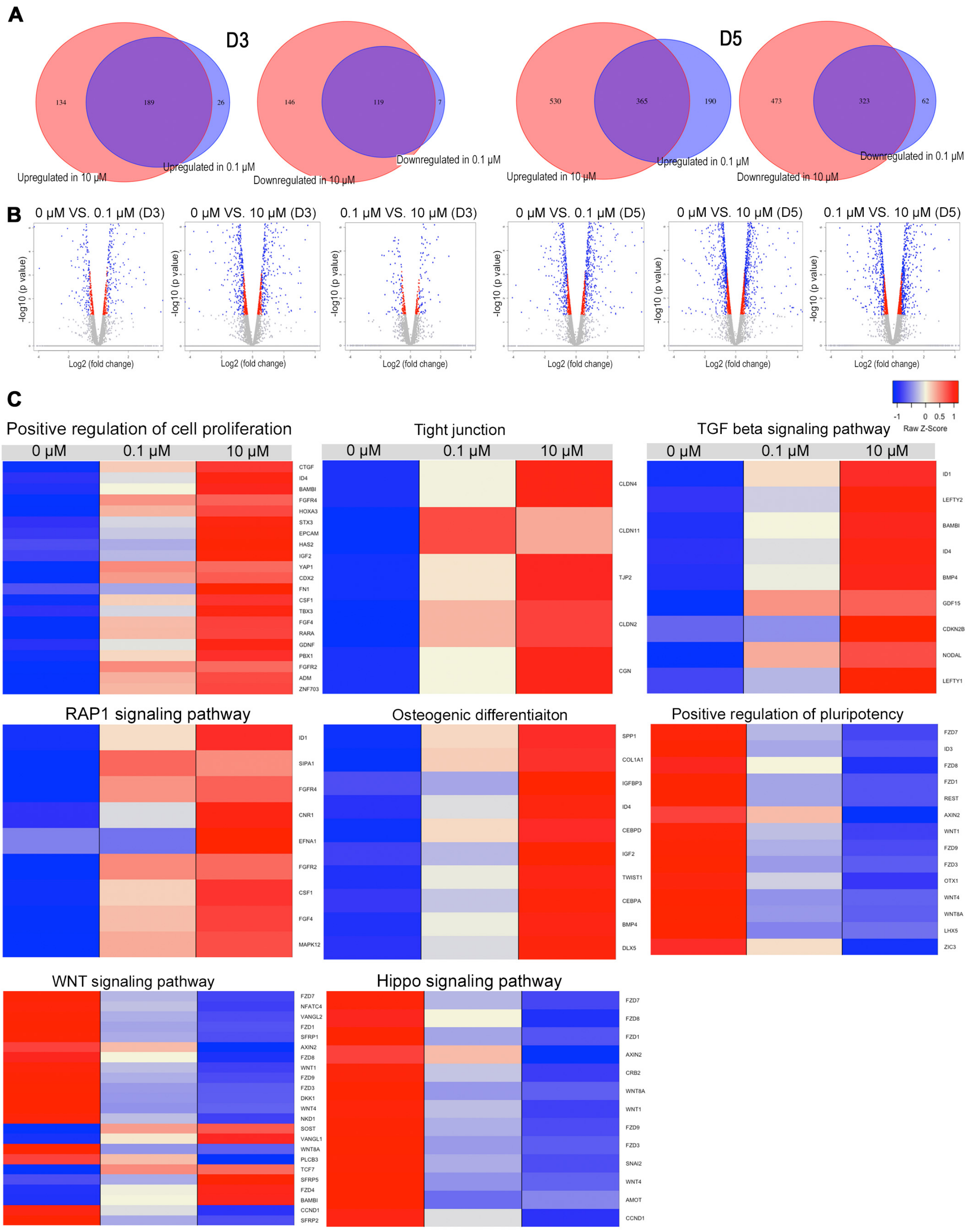

Supplementary Figure 6. Transcriptomic comparison of EBs treated with RA versus untreated EBs. (A) Venn diagram showing the number of the DEGs (upregulated and downregulated genes) in H9-hESC-derived EBs treated with 0.1 μM or 10 μM RA in

comparison to untreated EBs at day 3 (D3) and day 5 (D5) of differentiation. (B) Volcano plot of the DEGs between RA untreated and RA treated cells at D3 and D5. (C) Heatmaps showing DEGs in H9-hESC-derived EBs treated with 10 μM RA compared to those treated with 0.1 μM RA and untreated EBs at D3 of differentiation. The relative value for each gene is depicted by color intensity, with red indicating upregulated and blue indicating downregulated genes.

Supplementary Tables

Table S1: The details of the antibodies used for flow cytometry and immunostaining

For Flow Cytometry			
**Antibody**	**Company**	**Catalog #**	**Dilution or Concentration**
APC anti-mouse/human CD44	Biolegend	103012	1:100
APC anti-human CD73 (Ecto-5'-nucleotidase)	Biolegend	344006	1:100
APC anti-human CD90 (Thy1)	Biolegend	328114	1:100
APC anti-human CD105	Biolegend	323208	1:100
APC anti-human CD271 (NGFR)	Biolegend	345108	1:100
PE anti-human CD45	Biolegend	304008	1:100
FITC anti-human CD14	Biolegend	301804	1:100
FITC anti-human CD19	Biolegend	302206	1:100
FITC anti-human CD34	Biolegend	343504	1:100
Mouse anti-FABP4	Abcam	ab93945	1:100
Rabbit anti-Sox2	ThermoFisher Scientific	48-1400	1:100
Alexa Fluor 647 anti-mouse IgG	ThermoFisher Scientific	A31571	1:500
Alexa Fluor 488 anti-rabbit IgG	ThermoFisher Scientific	A21206	1:500
Mouse IgG1, κ Isotype Ctrl	Abcam	ab91353	1:100
APC Mouse IgG1, κ Isotype Ctrl	Biolegend	400122	1:100
APC Rat IgG2b, κ Isotype Ctrl	Biolegend	400612	1:100
PE Mouse IgG1, κ Isotype Ctrl	Biolegend	400114	1:100
FITC Mouse IgG2a, κ Isotype Ctrl	Biolegend	400208	1:100
FITC Mouse IgG1, κ Isotype Ctrl	Biolegend	400107	1:100
**For immunostaining**			
Rabbit anti-PPARγ	CST	2443	1:1000
Goat anti-FABP4	R & D Systems	AF3150	1:200
Mouse anti-adiponectin	Abcam	ab22554	1:400
Alexa Fluor 488 anti-goat IgG	ThermoFisher Scientific		1:500
Alexa Fluor 568 anti-rabbit IgG	ThermoFisher Scientific	A-10042	1:500
Alexa Fluor 568 anti-mouse IgG	ThermoFisher Scientific	A-10037	1:500

Table S2: The list of primers for qPCR used in this study

Gene name	Forward	Reverse	Product size
ISL1	CTGTGGACATTACTCCCTCTTAC	GCAACCAACACATAGGGAAATC	87
EPCAM	GGGGAACAACTGGATCTGGA	CAGCACAACAATTCCAGCAAC	150
SOX4	TCCTCTTCCTCCTCCTCG	TCGAGTTCCCGGACTACTGC	192
GATA6	AAGCGCGTGCCTTCATCA	TCATAGCAAGTGGTCTGGGC	157
KRT18	GTACTGGTCTCAGCAGATTG	CTGGCCTTCAGATTTCTCAT	150
BMP4	GACTACATGCGGGATCTTTAC	GGATGTTCTCCAGATGTTCTTC	148
HLA-C	TGATGTGTAGGAGGAAGAG	GTCTCAGGCTTTACAAGTG	113
CCDN1	GGCACAAGTCCTGGATGTTG	CCAGAAATGCACAGACCCAG	135
WNT1	CTGTCCTGCCTCCTCATC	GGACCCAGCACAATAAATAGTT	103
OCT4	GACAGGGGGAGGGGAGGAGCT AGG	CTTCCCTCCAACCAGTTGCCCCAA AC	119
NANOG	CATGAGTGTGGATCCAGCTTG	CCTGAATAAGCAGATCCATGG	192
GAPDH	ACGACCACTTTGTCAAGCTCAT TTC	GCAGTGAGGGTCTCTCTCTTCCTC T	132

Table S3: Top upregulated and downregulated genes at days 3 and 5 in cells treated with 0.1 μM and 10 μM RA compared with those treated with 0 μM (*p*<0.05).

Gene symbol	Gene title	0.1 μM RA vs. 0 μM		10 μM RA vs. 0 μM	
		Log2 (FC)	p-value	Log2 (FC)	p-value
**Downregulated genes at Day 3**					
OTX1	Orthodenticle Homeobox 1	-1.38	0.0234	-5.03	0.0038
SNAI2	Snail Family Transcriptional Repressor 2	-1.77	0.00135	-3.67	0.00055
DKK1	Dickkopf WNT Signaling Pathway Inhibitor 1	-1.93	0.0026	-2.87	0.0022
NKD1	Naked Cuticle Homolog 1	-1.31	0.00015	-2.75	5.00E-05
LHX5	LIM Homeobox 5	-2.34	0.0024	-2.69	0.00505
WNT4	Wnt family member 4	-1.46	0.0004	-1.85	0.0002
SFRP2	Secreted Frizzled Related Protein 2	-1.08	0.00015	-1.64	5.00E-05
SFRP1	Secreted Frizzled Related Protein 1	-0.97	0.0073	-1.45	5.00E-05
FZD1	Frizzled Class Receptor 1	-0.99	0.0032	-1.40	0.00035
FZD7	Frizzled Class Receptor 7	-0.80	0.013	-1.28	0.0002
WNT8A	Wnt Family Member 8A	-0.98	0.01295	-1.23	0.01045
AMOT	Angiomotin	-1.01	0.0016	-0.91	0.00655

**Downregulated genes at Day 5**					
WNT8A	Wnt Family Member 8A	-4.71	5.00E-05	-6.74	0.02465
LHX5	LIM Homeobox 5	-3.71	0.00135	-5.26	0.00945
CXCR4	C-X-C chemokine receptor type 4	-1.41	0.0001	-4.17	5.00E-05
NANOG	Homeobox Transcription Factor Nanog	-1.64	0.004	-4.17	0.00105
FZD10	Frizzled Class Receptor 10	-0.96	0.00185	-4.12	5.00E-05
EPHA4	EPH Receptor A4	-3.76	5.00E-05	-3.22	5.00E-05
CCND1	Cyclin D1	-1.59	5.00E-05	-3.13	5.00E-05
L1CAM	L1 Cell Adhesion Molecule)	-1.58	0.00015	-3.07	5.00E-05
NKD1	Naked Cuticle Homolog 1	-1.77	5.00E-05	-2.76	5.00E-05
ZIC3	Zic Family Member 3	-0.65	0.0245	-2.57	5.00E-05
LRRC4	Leucine Rich Repeat Containing 4	-1.11	0.0429	-2.49	5.00E-05
EPHA7	EPH Receptor A7	-1.84	5.00E-05	-2.16	5.00E-05
LEF1	Lymphoid Enhancer Binding Factor 1	-1.29	0.0006	-2.15	5.00E-05
SEMA5B	Semaphorin 5B	-0.86	0.0048	-2.10	5.00E-05
WNT5B	Wnt Family Member 5B	-1.95	5.00E-05	-1.97	5.00E-05
WNT10B	Wnt Family Member 10B	-0.83	0.03925	-1.93	5.00E-05
POU5F1	POU Class 5 Homeobox 1	-1.03	0.01535	-1.85	5.00E-05
SEMA3G	Semaphorin 3G	-1.27	0.01155	-1.81	0.00035
FZD7	Frizzled Class Receptor 7	-1.72	5.00E-05	-1.80	5.00E-05
FZD1	Frizzled Class Receptor 1	-1.51	5.00E-05	-1.76	5.00E-05
FZD3	Frizzled Class Receptor 3	-0.67	0.0239	-1.68	5.00E-05
EFNB1	Ephrin B1	-0.72	0.0145	-1.65	5.00E-05
WNT1	Wnt Family Member 1	-1.05	0.0029	-1.51	5.00E-05
NFATC4	Nuclear Factor Of Activated T Cells 4	-0.82	0.00595	-1.34	5.00E-05
SFRP2	Secreted Frizzled Related Protein 2	-1.55	5.00E-05	-1.26	5.00E-05
SFRP1	Secreted Frizzled Related Protein 1	-1.47	5.00E-05	-1.03	0.00015
SEMA7A	Semaphorin 7A	-1.05	0.0065	-0.93	0.0071
DKK1	Dickkopf WNT Signaling Pathway Inhibitor 1	-2.73	5.00E-05	-0.86	0.02085
ID3	Inhibitor Of DNA Binding 3, HLH Protein	-1.19	5.00E-05	-0.71	0.0147
**Upregulated genes at Day 3**					
LEFTY2	Left-Right Determination Factor 2	3.99	5.00E-05	5.40	5.00E-05
DUSP9	Dual Specificity Phosphatase 9	3.38	5.00E-05	4.67	5.00E-05
LEFTY1	Left-Right Determination Factor 1	2.40	0.00135	4.06	0.00015
FZD4	Frizzled Class Receptor 4	2.96	5.00E-05	3.93	5.00E-05
SFRP5	Secreted frizzled-related protein 5	1.75	0.01255	3.50	0.00015
DLX5	Distal-Less Homeobox 5	2.23	0.0001	3.34	5.00E-05
CNR1	Cannabinoid Receptor 1	1.88	5.00E-05	2.93	5.00E-05
TBX3	T-Box 3	1.85	0.0017	2.93	5.00E-05

SOST	Sclerostin	2.37	0.00985	2.59	0.003
FGF4	Fibroblast Growth Factor 4	1.65	0.02975	2.01	0.01495
HAND1	Heart And Neural Crest Derivatives Expressed 1	1.99	0.00295	1.98	0.0069
SKIL	SKI Like Proto-Oncogene	1.51	5.00E-05	1.92	5.00E-05
BAMBI	BMP And Activin Membrane Bound Inhibitor	1.20	0.00015	1.86	5.00E-05
ID1	Inhibitor Of DNA Binding 1, HLH Protein	1.12	0.00035	1.58	5.00E-05
FGFR4	Fibroblast Growth Factor Receptor 4	1.43	0.0005	1.56	0.0001
BMP4	Bone Morphogenetic Protein 4	0.78	0.03155	1.35	0.00085
MAPK12	Mitogen-Activated Protein Kinase 12	1.04	0.00295	1.23	0.0011
CSF1	Colony Stimulating Factor 1	1.88	5.00E-05	1.16	0.00805
BMI1	BMI1 Proto-Oncogene, Polycomb Ring Finger	1.12	0.0006	1.02	0.00465
**Upregulated genes at Day 5**					
LEFTY2	Left-Right Determination Factor 2	8.61	5.00E-05	9.92	5.00E-05
CLDN2	Claudin 2	7.83	0.0013	6.48	0.00365
IGFBP3	Insulin-like growth factor-binding protein 3	4.01	5.00E-05	5.48	5.00E-05
CDKN2B	Cyclin Dependent Kinase Inhibitor 2B	2.22	0.0025	5.30	5.00E-05
CLDN4	Claudin 4	3.31	5.00E-05	4.74	5.00E-05
GDF6	Growth Differentiation Factor 6	2.80	5.00E-05	4.73	5.00E-05
LEFTY1	Left-Right Determination Factor 1	2.58	5.00E-05	4.05	5.00E-05
CLDN1	Claudin 1	5.35	5.00E-05	3.89	5.00E-05
FZD4	Frizzled Class Receptor 4	2.44	0.00015	3.36	5.00E-05
CDH1	Cadherin 1	1.52	5.00E-05	3.19	5.00E-05
PITX2	Paired Like Homeodomain 2	2.12	5.00E-05	3.12	5.00E-05
CLDN3	Claudin 3	1.74	0.0007	2.61	5.00E-05
BAMBI	BMP And Activin Membrane Bound Inhibitor	0.92	0.00515	2.07	5.00E-05
ID1	Inhibitor Of DNA Binding 1, HLH Protein	1.08	0.00095	1.94	5.00E-05
BMP4	Bone Morphogenetic Protein 4	1.179	0.00065	1.75	5.00E-05
SDC4	Syndecan 4	0.75	0.018	1.66	5.00E-05
TEAD3	TEA Domain Transcription Factor 3	0.91	0.00455	1.55	5.00E-05
PERP	P53 Apoptosis Effector Related To PMP22	0.85138	0.0047	1.45	5.00E-05
GADD45A	Growth Arrest And DNA Damage Inducible Alpha	0.97	0.00325	1.30	5.00E-05
PTPRF	Protein Tyrosine Phosphatase Receptor Type F	0.58	0.04935	1.26	5.00E-05
CDK6	Cyclin Dependent Kinase 6	0.89	0.0033	1.20	5.00E-05
FZD5	Frizzled Class Receptor 5	1.25	0.00175	1.169	0.0017
ALCAM	Activated Leukocyte Cell Adhesion Molecule	1.02	0.00075	0.77	0.00895

Table S4. Enriched functions in the upregulated differentially expressed genes (DEGs) in day 5-old EBs treated with 10 µM RA compared with those untreated (p <0.05). n: number of genes from the DEG list in each specific annotation term.

Gene symbol	Gene title	10 µM RA vs. 0 µM	
		Log2 (FC)	p-value
**Positive regulation of cell proliferation (n=39)**			
BNC1	basonuclin 1	4.88	5.00E-05
ISL1	ISL LIM homeobox 1	4.71	5.00E-05
TBX3	T-box 3	4.21	5.00E-05
IGF2	insulin like growth factor 2	4.10	5.00E-05
CCKBR	cholecystokinin B receptor	3.83	5.00E-05
HOXA3	homeobox A3	3.67	5.00E-05
EPCAM	epithelial cell adhesion molecule	3.67	5.00E-05
KLF5	Kruppel like factor 5	3.39	5.00E-05
RAB25	RAB25, member RAS oncogene family	2.89	5.00E-05
FN1	fibronectin 1	2.82	5.00E-05
EDN1	endothelin 1	2.79	0.00055
ZNF703	zinc finger protein 703	2.72	5.00E-05
NKX3-1	NK3 homeobox 1	2.47	0.0026
FGFR4	fibroblast growth factor receptor 4	2.28	5.00E-05
TBX2	T-box 2	2.24	0.0007
PDGFRA	platelet derived growth factor receptor alpha	2.12	5.00E-05
BAMBI	BMP and activin membrane bound inhibitor	2.07	5.00E-05
SP6	Sp6 transcription factor	1.90	5.00E-05
HAS2	hyaluronan synthase 2	1.88	5.00E-05
PTPN6	protein tyrosine phosphatase, non-receptor type 6	1.78	5.00E-05
EGR4	early growth response 4	1.67	0.0293
GAB2	GRB2 associated binding protein 2	1.65	5.00E-05
CTF1	cardiotrophin 1	1.64	0.00045
NCCRP1	non-specific cytotoxic cell receptor protein 1 homolog	1.62	0.00315
LIF	leukemia inhibitory factor	1.50	0.00155
CLCF1	cardiotrophin-like cytokine factor 1	1.47	0.02435
TNC	tenascin C	1.41	5.00E-05
RARA	retinoic acid receptor alpha	1.37	5.00E-05
TGFB1	transforming growth factor beta 1	1.36	0.00015
FOSL1	FOS like 1, AP-1 transcription factor subunit	1.30	0.005
SOX4	SRY-box 4	1.29	5.00E-05
PBX1	PBX homeobox 1	1.29	5.00E-05

SPHK2	sphingosine kinase 2	1.23	0.03345
STX3	syntaxin 3	1.22	5.00E-05
IL6ST	interleukin 6 signal transducer	1.06	0.001
EPHA1	EPH receptor A1	1.04	0.0025
CAPN1	calpain 1	0.97	0.00165
ITGAV	integrin subunit alpha V	0.67	0.0231
THBS1	thrombospondin 1	0.66	0.0302
**Negative regulation of apoptotic process (n=34)**			
SLC40A1	solute carrier family 40 member 1	6.63	0.0232
GATA6	GATA binding protein 6	5.44	5.00E-05
OSR1	odd-skipped related transciption factor 1	4.82	5.00E-05
TBX3	T-box 3	4.21	5.00E-05
KRT18	keratin 18	3.99	5.00E-05
TWIST1	twist family bHLH transcription factor 1	3.95	5.00E-05
EPCAM	epithelial cell adhesion molecule	3.67	5.00E-05
MMP9	matrix metallopeptidase 9	3.21	0.0012
PLK2	polo like kinase 2	3.09	5.00E-05
ANXA1	annexin A1	2.89	5.00E-05
ID1	inhibitor of DNA binding 1, HLH protein	2.52	5.00E-05
SMAD6	SMAD family member 6	2.35	5.00E-05
DAB2	DAB2, clathrin adaptor protein	2.31	5.00E-05
SERPINB9	serpin family B member 9	1.91	5.00E-05
BMP4	bone morphogenetic protein 4	1.75	5.00E-05
RARA	retinoic acid receptor alpha	1.37	5.00E-05
CYR61	cysteine rich angiogenic inducer 61	1.30	5.00E-05
EGR3	early growth response 3	1.27	0.0037
SPHK2	sphingosine kinase 2	1.23	0.03345
NFKBIA	NFKB inhibitor alpha	1.20	0.0008
SOCS3	suppressor of cytokine signaling 3	1.18	0.0004
TFAP2A	transcription factor AP-2 alpha	1.14	0.0001
CDKN1A	cyclin dependent kinase inhibitor 1A	1.08	0.0028
GAS6	growth arrest specific 6	1.07	0.00355
IL6ST	interleukin 6 signal transducer	1.06	0.001
PRNP	prion protein	1.06	0.0014
SQSTM1	sequestosome 1	1.05	0.007
BAG3	BCL2 associated athanogene 3	1.04	0.00165
SIAH2	siah E3 ubiquitin protein ligase 2	0.83	0.0145
CD74	CD74 molecule	0.82	0.0203
PIM1	Pim-1 proto-oncogene, serine/threonine kinase	0.81	0.00475

VEGFB	vascular endothelial growth factor B	0.74	0.03475
ACTC1	actin, alpha, cardiac muscle 1	0.73	0.02365
THBS1	thrombospondin 1	0.66	0.0302
**Cell adhesion molecules (CAMs) (n=19)**			
CLDN2	claudin 2	6.48	0.00365
CDH5	cadherin 5	4.93	5.00E-05
CLDN4	claudin 4	4.74	5.00E-05
CLDN1	claudin 1	3.89	5.00E-05
CDH1	cadherin 1	3.19	5.00E-05
CDH3	cadherin 3	2.82	5.00E-05
CLDN3	claudin 3	2.60	5.00E-05
CLDN23	claudin 23	1.91	0.00295
SDC4	syndecan 4	1.66	5.00E-05
CLDN9	claudin 9	1.59	0.02635
PTPRF	protein tyrosine phosphatase, receptor type F	1.26	5.00E-05
HLA-DRB1	major histocompatibility complex, class II, DR beta 1	1.24	0.00395
CLDN6	claudin 6	1.17	0.00015
HLA-C	major histocompatibility complex, class I, C	1.11	0.0049
F11R	F11 receptor	1.11	0.00015
MPZL1	myelin protein zero like 1	0.98	0.00095
NECTIN2	nectin cell adhesion molecule 2	0.79	0.00655
ALCAM	activated leukocyte cell adhesion molecule	0.77	0.00895
ITGAV	integrin subunit alpha V	0.67	0.0231
**Tight junction (n=14)**			
CLDN2	claudin 2	6.48	0.00365
CLDN4	claudin 4	4.74	5.00E-05
CLDN1	claudin 1	3.88	5.00E-05
CRB3	crumbs 3, cell polarity complex component	3.76	0.01345
CLDN3	claudin 3	2.60	5.00E-05
LLGL2	LLGL2, scribble cell polarity complex component	2.52	5.00E-05
CGN	cingulin	2.29	5.00E-05
CLDN23	claudin 23	1.91	0.00295
CLDN9	claudin 9	1.59	0.02635
TJP2	tight junction protein 2	1.55	5.00E-05
CLDN6	claudin 6	1.17	0.00015
F11R	F11 receptor	1.11	0.00015
MYL12B	myosin light chain 12B	1.10	0.00705
PARD6B	par-6 family cell polarity regulator beta	0.89	0.00965
**ECM-receptor interaction (n=13)**			

SPP1	secreted phosphoprotein 1	3.45	5.00E-05
FN1	fibronectin 1	2.82	5.00E-05
ITGA3	integrin subunit alpha 3	2.74	5.00E-05
SDC4	syndecan 4	1.66	5.00E-05
COL1A1	collagen type I alpha 1 chain	1.62	5.00E-05
TNC	tenascin C	1.41	5.00E-05
LAMA5	laminin subunit alpha 5	1.37	5.00E-05
LAMB1	laminin subunit beta 1	1.28	5.00E-05
HSPG2	heparan sulfate proteoglycan 2	0.96	0.0052
DAG1	dystroglycan 1	0.78	0.00595
ITGAV	integrin subunit alpha V	0.67	0.0231
THBS1	thrombospondin 1	0.66	0.0302
SV2A	synaptic vesicle glycoprotein 2A	0.60	0.03965
**Focal adhesion (n=17)**			
SPP1	secreted phosphoprotein 1	3.45	5.00E-05
MYL7	myosin light chain 7	3.19	0.02435
FN1	fibronectin 1	2.82	5.00E-05
ITGA3	integrin subunit alpha 3	2.74	5.00E-05
PDGFRA	platelet derived growth factor receptor alpha	2.12	5.00E-05
COL1A1	collagen type I alpha 1 chain	1.62	5.00E-05
FLNB	filamin B	1.47	5.00E-05
FLNC	filamin C	1.45	5.00E-05
TNC	tenascin C	1.41	5.00E-05
LAMA5	laminin subunit alpha 5	1.37	5.00E-05
LAMB1	laminin subunit beta 1	1.28	5.00E-05
MYL12B	myosin light chain 12B	1.10	0.00705
BCAR1	BCAR1, Cas family scaffolding protein	0.97	0.00145
JUN	Jun proto-oncogene, AP-1 transcription factor subunit	0.91	0.0042
VEGFB	vascular endothelial growth factor B	0.74	0.03475
ITGAV	integrin subunit alpha V	0.67	0.0231
THBS1	thrombospondin 1	0.66	0.0302
**Hippo signaling pathway (n=15)**			
GDF6	growth differentiation factor 6	4.73	5.00E-05
WNT6	Wnt family member 6	3.77	5.00E-05
FZD4	frizzled class receptor 4	3.36	5.00E-05
CDH1	cadherin 1	3.19	5.00E-05
LLGL2	LLGL2, scribble cell polarity complex component	2.52	5.00E-05
ID1	inhibitor of DNA binding 1, HLH protein	1.94	5.00E-05
AMOT	angiomotin	1.75	5.00E-05

BMP4	bone morphogenetic protein 4	1.75	5.00E-05
TEAD3	TEA domain transcription factor 3	1.55	5.00E-05
FZD8	frizzled class receptor 8	1.50	5.00E-05
TGFB1	transforming growth factor beta 1	1.36	0.00015
FZD5	frizzled class receptor 5	1.17	0.0017
SERPINE1	serpin family E member 1	0.94	0.0118
PARD6B	par-6 family cell polarity regulator beta	0.89	0.00965
DLG3	discs large MAGUK scaffold protein 3	0.60	0.04
**TGF-beta signaling pathway (n=14)**			
LEFTY2	left-right determination factor 2	9.92	5.00E-05
CDKN2B	cyclin dependent kinase inhibitor 2B	5.30	5.00E-05
GDF6	growth differentiation factor 6	4.73	5.00E-05
LEFTY1	left-right determination factor 1	4.05	5.00E-05
AMHR2	anti-Mullerian hormone receptor type 2	3.34	0.001
PITX2	paired like homeodomain 2	3.12	5.00E-05
SMAD6	SMAD family member 6	2.35	5.00E-05
NODAL	nodal growth differentiation factor	2.33	5.00E-05
BAMBI	BMP and activin membrane bound inhibitor	2.07	5.00E-05
ID1	inhibitor of DNA binding 1, HLH protein	1.94	5.00E-05
BMP4	bone morphogenetic protein 4	1.75	5.00E-05
TGFB1	transforming growth factor beta 1	1.36	0.00015
SMAD7	SMAD family member 7	1.12	0.00035
THBS1	thrombospondin 1	0.66	0.0302
**Positive regulation of epithelial mesenchymal transition (n=7)**			
TWIST1	twist family bHLH transcription factor 1	3.95	5.00E-05
ZNF703	zinc finger protein 703	2.73	5.00E-05
DAB2	DAB2, clathrin adaptor protein	2.31	5.00E-05
BAMBI	BMP and activin membrane bound inhibitor	2.07	5.00E-05
COL1A1	collagen type I alpha 1 chain	1.63	5.00E-05
TGFB1	transforming growth factor beta 1	1.36	0.00015
BCL9L	B-cell CLL/lymphoma 9-like	0.92	0.00155

Table S5. Enriched functions in the downregulated differentially expressed genes (DEGs) in day 5-old EBs treated with 10 µM RA compared with those untreated (*p* <0.05). n: number of genes from the DEG list in each specific annotation term.

Gene symbol	Gene title	10 µM RA vs. 0 µM	
		Log2 (FC)	p-value
**WNT signaling pathway (n=33)**			
WNT8A	Wnt family member 8A	-6.74	0.02465
FZD10	frizzled class receptor 10	-4.11	5.00E-05
WNT3A	Wnt family member 3A	-3.16	5.00E-05
CCND1	cyclin D1	-3.13	5.00E-05
NKD1	naked cuticle homolog 1	-2.76	5.00E-05
ROR1	Receptor Tyrosine Kinase Like Orphan Receptor 1	-2.35	5.00E-05
LEF1	lymphoid enhancer binding factor 1	-2.15	5.00E-05
APCDD1	Adenomatosis Polyposis Coli Down-Regulated 1 Protein	-2.04	5.00E-05
LRP4	LDL Receptor Related Protein 4	-2.01	0.0004
FRZB	Secreted Frizzled-Related Protein 3	-2.01	5.00E-05
WNT5B	Wnt family member 5B(WNT5B)	-1.97	5.00E-05
WNT10B	Wnt family member 10B	-1.92	5.00E-05
NOTUM	Notum, Palmitoleoyl-Protein Carboxylesterase	-1.92	5.00E-05
FZD7	frizzled class receptor 7	-1.80	5.00E-05
FZD1	frizzled class receptor 1	-1.76	5.00E-05
GPC4	glypican 4	-1.69	5.00E-05
FZD3	frizzled class receptor 3	-1.68	5.00E-05
AXIN2	axin 2	-1.61	5.00E-05
WNT1	Wnt family member 1	-1.51	5.00E-05
RSPO3	R-Spondin 3	-1.50	5.00E-05
TLE4	TLE Family Member 4, Transcriptional Corepressor	-1.49	5.00E-05
NFATC4	nuclear factor of activated T-cells 4	-1.33	5.00E-05
FZD9	frizzled class receptor 9	-1.32	0.00775
SFRP2	secreted frizzled related protein 2	-1.26	5.00E-05
VANGL2	VANGL planar cell polarity protein 2	-1.21	5.00E-05
SFRP1	secreted frizzled related protein 1	-1.03	0.00015
DKK1	dickkopf WNT signaling pathway inhibitor 1	-0.86	0.02085
ROR2	Receptor Tyrosine Kinase Like Orphan Receptor 2	-0.85	0.0039
MYC	v-myc avian myelocytomatosis viral oncogene homolog	-0.77	0.0195
RAC3	ras-related C3 botulinum toxin substrate 3	-0.69	0.04325
CTNNBIP1	catenin beta interacting protein 1	-0.62	0.03805
CCND2	cyclin D2	-0.61	0.02845
LRP6	LDL receptor related protein 6	-0.60	0.0381

**Neuronal differentiation and axonal guidance (n=34)**			
CXCR4	C-X-C motif chemokine receptor 4	-4.17	5.00E-05
EPHA4	EPH receptor A4	-3.21	5.00E-05
L1CAM	L1 cell adhesion molecule	-3.07	5.00E-05
SHANK1	SH3 And Multiple Ankyrin Repeat Domains 1	-2.88	5.00E-05
FEZF1	FEZ Family Zinc Finger 1	-2.74	0.0017
ZIC2	Zic Family Member 2	-2.64	5.00E-05
ZIC3	Zic Family Member 3	-2.57	5.00E-05
LRRC4	leucine rich repeat containing 4	-2.49	5.00E-05
GPM6B	Glycoprotein M6B	-2.37	5.00E-05
ZIC1	Zic Family Member 1	-2.23	5.00E-05
EPHA7	EPH receptor A7	-2.16	5.00E-05
NGFR	Nerve Growth Factor Receptor	-2.16	5.00E-05
ZIC5	Zic Family Member 5	-2.16	5.00E-05
SEMA5B	semaphorin 5B	-2.10	5.00E-05
ASCL1	Achaete-Scute Family BHLH Transcription Factor 1	-1.88	0.00735
SEMA3G	semaphorin 3G	-1.81	0.00035
PAX3	*Paired Box 3*	-1.74	5.00E-05
EFNB1	Ephrin B1	-1.65	5.00E-05
MDGA1	MAM Domain Containing Glycosylphosphatidylinositol Anchor 1	-1.50	5.00E-05
SEMA3E	semaphorin 3E	-1.44	5.00E-05
NFATC4	Nuclear Factor Of Activated T Cells 4	-1.33	5.00E-05
SEMA4F	ssemaphorin 4F	-1.09	0.00335
SOX11	SRY-Box Transcription Factor 11	-1.02	0.00035
GAP43	G protein subunit alpha i2	-0.97	0.0012
KDM7A	Lysine Demethylase 7A	-0.95	0.0029
FYN	FYN proto-oncogene, Src family tyrosine kinase	-0.95	0.00165
SEMA7A	semaphorin 7A	-0.92	0.0071
EFNB3	Ephrin B3	-0.90	0.0031
DPYSL2	dihydropyrimidinase like 2	-0.89	0.0028
EFNA3	ephrin A3	-0.85	0.0201
SEMA3C	semaphorin 3C	-0.85	0.00475
TRNP1	TMF1 Regulated Nuclear Protein 1	-0.81	0.04625
ROBO3	*Roundabout Guidance Receptor 3*	-0.78	0.0431
**Signaling pathways regulating pluripotency of stem cells (n=23)**			
LHX5	LIM homeobox 5	-5.26	0.00945
NANOG	Nanog homeobox	-4.17	0.00105
WNT8A	Wnt family member 8A	-6.74	0.02465
FZD10	frizzled class receptor 10	-4.11	5.00E-05

WNT3A	Wnt family member 3A	-3.15	5.00E-05
ZIC3	Zic Family Member 3	-2.57	5.00E-05
SOX2	SRY-box 2	-2.15	5.00E-05
WNT5B	Wnt family member 5B	-1.97	5.00E-05
WNT10B	Wnt family member 10B	-1.92	5.00E-05
POU5F1	POU class 5 homeobox 1	-1.84	5.00E-05
FZD7	frizzled class receptor 7	-1.80	5.00E-05
FZD1	frizzled class receptor 1	-1.76	5.00E-05
FZD3	frizzled class receptor 3	-1.68	5.00E-05
AXIN2	axin 2	-1.61	5.00E-05
WNT1	Wnt family member 1	-1.51	5.00E-05
FZD9	frizzled class receptor 9	-1.32	0.00775
INHBE	inhibin beta E subunit	-1.28	0.04045
ACVR2B	activin A receptor type 2B	-0.79	0.00515
MYC	v-myc avian myelocytomatosis viral oncogene homolog	-0.77	0.0195
FGFR3	fibroblast growth factor receptor 3	-0.73	0.0152
ID3	inhibitor of DNA binding 3, HLH protein	-0.71	0.0147
RIF1	replication timing regulatory factor 1	-0.61	0.03265
REST	RE1 silencing transcription factor	-0.60	0.03235
**Hippo signaling pathway (n=22)**			
WNT8A	Wnt family member 8A	-6.73	0.02465
SNAI2	snail family transcriptional repressor 2	-4.73	5.00E-05
FZD10	frizzled class receptor 10	-4.11	5.00E-05
GDF7	growth differentiation factor 7	-3.32	5.00E-05
WNT3A	Wnt family member 3A	-3.15	5.00E-05
CCND1	cyclin D1	-3.13	5.00E-05
SOX2	SRY-box 2	-2.15	5.00E-05
LEF1	lymphoid enhancer binding factor 1	-2.14	5.00E-05
WNT5B	Wnt family member 5B	-1.97	5.00E-05
WNT10B	Wnt family member 10B	-1.92	5.00E-05
FZD7	frizzled class receptor 7	-1.79	5.00E-05
FZD1	frizzled class receptor 1	-1.76	5.00E-05
FZD3	frizzled class receptor 3	-1.68	5.00E-05
AXIN2	axin 2	-1.60	5.00E-05
DLG4	discs large MAGUK scaffold protein 4	-1.57	0.00885
WNT1	Wnt family member 1	-1.51	5.00E-05
FZD9	frizzled class receptor 9	-1.32	0.00775
TGFBR1	*Transforming Growth Factor Beta Receptor 1*	-0.98	0.001
MYC	v-myc avian myelocytomatosis viral oncogene homolog	-0.77	0.0195

TEAD1	TEA domain transcription factor 1	-0.67	0.01905
GLI2	GLI family zinc finger 2	-0.63	0.03465
CCND2	cyclin D2	-0.61	0.02845
**RAP1 signaling pathway (n=14)**			
FGF3	fibroblast growth factor 3	-6.11	0.00325
FGF4	fibroblast growth factor 4	-4.68	0.00325
FGF19	fibroblast growth factor 19	-2.67	5.00E-05
NGFR	nerve growth factor receptor	-2.16	5.00E-05
LPAR4	lysophosphatidic acid receptor 4	-2.149	5.00E-05
FGF11	fibroblast growth factor 11	-1.75	5.00E-05
VEGFA	vascular endothelial growth factor A	-1.72	5.00E-05
ARAP3	ArfGAP with RhoGAP domain, ankyrin repeat and PH domain 3	-1.50	0.0002
P2RY1	purinergic receptor P2Y1	-1.26	0.00055
EFNA3	ephrin A3	-0.85	0.0201
FGFR3	fibroblast growth factor receptor 3	-0.73	0.0152
GNAI2	G protein subunit alpha i2	-0.71	0.0129
RAC3	ras-related C3 botulinum toxin substrate 3	-0.69	0.04325
GNAS	GNAS complex locus	-0.65	0.0203

Table S6. RNA sequencing data for MSCs obtained from H9 hESCs without or with 10 µM RA treatment. Genes encoding factors that mediate the immunoregulatory and paracrine functions of MSCs are in black. Genes encoding receptors for pro-inflammatory factors that are involved in MSC activation are in blue.

Gene	Locus	0 µM_FPKM	10 µM_FPKM	log2 (FC)	p-value	q-value	Encoded protein
TMSB4X	chrX:12975106-12977227	717.923	465.854	-0.62395	0.0766	0.885356	TB4
LGALS1	chr22:37668025-38041915	18.8764	12.7176	-0.56976	0.6595	0.9998	Galectin-1
TGFB1	chr19:41330322-41353883	8.07926	10.0063	0.30862	0.3165	0.9998	TGF-β1
TNFRSF1A	chr12:6328756-6342117	8.17131	9.87805	0.27366	0.3715	0.9998	TNF-α receptor
VEGFB	chr11:64234583-64239264	9.77189	8.96304	-0.12465	0.6988	0.9998	VEGFB
VEGFA	chr6:43770208-43786486	2.00835	4.80677	1.25906	0.0002	0.011223	VEGFA
CCL2	chr17:34255276-34257201	3.05154	2.14995	-0.5052	0.3762	0.9998	CCL2
CD274	chr9:5450502-5470567	1.55175	1.96088	0.33761	0.32295	0.9998	PDL1
BMP4	chr14:53949735-53956891	2.04656	0.848991	-1.26938	0.00885	0.216134	BMP4
IFNGR1	chr6:137197483-137219430	1.20325	0.933978	-0.36547	0.28765	0.9998	IFN-γ receptor
PDCD1LG2	chr9:5510544-5571282	0.325356	0.220826	-0.559	1	1	PDL2
IL6	chr7:22725394-22732002	0.0788886	0.0426081	-0.8887	1	1	IL6
HGF	chr7:81699007-81770198	0.0209038	0.0247064	0.24112	1	1	HGF
TNFAIP6	chr2:151337825-151380048	0.0175987	0.102173	2.53747	1	1	TSG6
IL10	chr1:206767602-206772494	0.00712189	0.0194796	1.4516	1	1	IL10
IDO1	chr8:39913808-39928791	0.00241028	0.00180885	-0.4141	1	1	IDO1
IDO2	chr8:39934954-40016391	0.000848992	0.00032889	-1.368	1	1	IDO2
FASLG	chr1:172659007-172666872	0	0	0	1	1	FASL
HLA-G	chr6:29826978-29831122	0	0	0	1	1	HLA-G
